# Inappropriate Sinus Tachycardia in Athletes: Could Nutraceuticals Play a Role?

**DOI:** 10.3390/jcdd12020073

**Published:** 2025-02-15

**Authors:** Antonio Scarà, Alessio Borrelli, Antonio Gianluca Robles, Sara Burazor, Lorenzo-Lupo Dei, Federico Zanin, Leonardo Pignalosa, Elena Cavarretta, Liuba Fusco, Andrej Pernat, Valerio Sanguigni, Silvio Romano, Luigi Sciarra

**Affiliations:** 1 San Carlo di Nancy-GVM Hospital, 00165 Rome, Italy; alessio.borrelli@libero.it (A.B.); federicozanin89@gmail.com (F.Z.); leonardopignalosa@hotmail.com (L.P.); 2 Department of Cardiovascular Disease, University of L’Aquila, 67100 L’Aquila, Italy; gianlucarobles24@gmail.com (A.G.R.); sara.burazor@student.univaq.it (S.B.); lorenzolupo.dei@graduate.univaq.it (L.-L.D.); silvio.romano@univaq.it (S.R.); luigi.sciarra@univaq.it (L.S.); 3 Department of Medico-Surgical Sciences and Biotechnologies, Sapienza University, 04100 Latina, Italy; elena.cavarretta@uniroma1.it; 4 Cardiology Department, Chelsea and Westminster Hospital, London SW10 9NH, UK; liubafusco@gmail.com; 5 Department of Cardiology, University Medical Centre Ljubljana, 1000 Ljubljana, Slovenia; andrej.pernat@mf.uni-lj.si; 6 Department of Internal Medicine, University of Rome “Tor Vergata”, 00133 Rome, Italy; sanguigni.valerio@gmail.com

**Keywords:** inappropriate sinus tachycardia, food supplement, ivabradine, Holter ECG, Herat rate, symptom relief

## Abstract

*Introduction:* Inappropriate sinus tachycardia (IST) is a syndrome characterized by unexpectedly fast and prolonged sinus rates at rest or with minimal physical activity. Epidemiologic characteristics are uncertain, but most patients are young and female. When IST occurs in athletes, its management (controlling symptoms and reducing heart rate) can present additional challenges. We designed an observational pilot study to investigate whether a food supplement can be useful in the treatment of IST when standard therapy is refused. *Methods:* We enrolled 50 consecutive recreational athletes affected by frequent recurrences of IST. Twelve-lead ECG and Holter ECG parameters were recorded at enrollment (T0) and after a 6-month treatment (T1) with the food supplement. Symptoms and quality of life were also evaluated through specific questionnaires. The study population was compared to a historical control group of 25 patients receiving ivabradine as treatment for the same clinical condition. *Results:* The resting ECG heart rate was 88.7 ± 12.4 bpm (T0) and 73.6 ± 6.6 bpm (T1) (*p* < 0.00001); Holter average heart rate was 88.4 ± 3.3 bpm and 74.9 ± 4.8 bpm (*p* < 0.0001). Holter ECG maximum heart rate was 147.1 ± 16.7 bpm and 139.2 ± 16.8 bpm (*p* = 0.06); Holter minimum heart rate was 49.9 ± 6.5 bpm and 50.5 ± 6.9 bpm (*p* = 0.33). Finally, the number of sustained episodes decreased from 3.3 ± 1.7 to 0.8 ± 0.8 (*p* < 0.00001). The following variations in ASTA scores were observed: ASTA symptom scale (range: 0–27) decreased from 14.9 ± 2.1 to 5.8 ± 1.4 (*p* < 0.00001), while ASTA HR QoL (range: 0–39) decreased from 24.1 ± 2.1 to 10.8 ± 2.3 (*p* < 0.00001). *Conclusions:* The findings of our pilot study suggest that this food supplement could play a beneficial role in managing symptoms and improving quality of life in recreational athletes affected by IST who refuse standard medical therapy. These clinical effects appear to correlate with significant improvements in resting ECG parameters and some Holter ECG parameters.

## 1. Introduction

Inappropriate sinus tachycardia (IST) is described as a syndrome characterized by unexpectedly fast sinus rates at rest or during minimal physical activity [1]. It can be associated with a wide range of symptoms, including palpitations, weakness, fatigue, dizziness, and near syncope. Moreover, heart rate acceleration in response to minimal exercise may be exaggerated, and heart rate recovery may be prolonged.

Sinus tachycardia, even when excessively fast, is generally a transient and reversible condition with an identifiable cause, such as caffeine ingestion, anxiety, or deconditioning, with a heart rate appropriate for the circumstance [1]. IST, however, is a more chronic issue that is not easily explained. No specific heart rate defines IST, but patients with IST typically have resting daytime sinus rates greater than 100 beats per minute and average 24 h heart rates greater than 90 beats per minute, which are not explained by physiological demands or conditions known to increase heart rate.

Epidemiological characteristics are uncertain, but most patients are young and female [2]. Episodes tend to appear abruptly and persist over months or years, though the natural cause remains unclear. The prognosis is generally benign. Although IST patients have faster heart rates, the rate slows somewhat during sleep and follows various diurnal patterns [2]. IST is rarely associated with tachycardia-induced cardiomyopathy, although isolated reports exist [3,4,5].

Managing IST—controlling symptoms and reducing heart rate—remains challenging, particularly due to the heterogeneity of clinical presentations and the sometimes-debilitating nature of the symptoms. Heart rate control, however, does not always eliminate symptoms. Various treatment options have been proposed for patients with IST, but these therapies are not always supported by strong scientific evidence. Beta-adrenergic blockers, even at high doses, are generally of limited effectiveness and tend to be associated with poorly tolerated side effects. Ivabradine may precipitate bradycardia (especially in combination with beta-blockers or calcium-channel blockers) and can cause headaches or phosphenes [6]. Other pharmacological treatments, such as fludrocortisone, volume expanders, pressure stockings, phenobarbital, clonidine, psychiatric support, and erythropoietin, have been proposed, but their efficacy appears limited and lacks strong scientific backing [7].

The treatment of IST presents additional challenges when it occurs in athletes due to factors such as possible sinus bradycardia beyond the arrhythmic episodes and the reluctance to use standard drugs.

A novel food supplement (SPINOFF^®^), containing predetermined amounts of linden, hawthorn, vitamin B1, and melatonin, has recently been investigated for the management of mild to moderate insomnia. It has shown positive effects on various aspects of sleep quality and psychological well-being [8]. Vitamin B1 is essential for the growth, development, and function of cells, as well as for normal brain, nerve, and heart function [9]. Melatonin is the primary hormone regulating the sleep–wake cycle and, according to European guidelines, helps reduce sleep onset latency [10]. Several studies have demonstrated sedative and anxiolytic effects of various linden (Tilia) species [11]. Hawthorn preparations are commonly used to treat angina, hypertension, arrhythmias, and congestive heart failure [12].

Based on the anxiolytic and potential antiarrhythmic effects of its components, we designed an observational pilot study to investigate the potential role of this food supplement in treating athletes suffering from inappropriate sinus tachycardia syndrome.

## 2. Materials and Methods

The present study enrolled 50 consecutive recreational athletes, from May 2023 to May 2024, affected by frequent recurrences (almost daily) of inappropriate sinus tachycardia who refused standard medical treatments. The sports practiced by the study population were running (15/50), swimming (10/50), padel/tennis (16/50), and cycling (9/50). The average weekly sports practice time was 3 ± 0.5 h.

Diagnosis was based on clinical presentation, a 12-lead electrocardiogram (ECG) at rest, and 24 h Holter ECG monitoring. Having enrolled recreational athletes in the study and taking into account their potential underlying vagal hypertonia, the diagnosis of inappropriate sinus tachycardia was primarily based on the presence of symptomatic episodes of sinus tachycardia, not attributable to physiological or functional conditions. A baseline echocardiogram was performed to rule out any ventricular or valvular structural abnormalities.

The following Holter ECG parameters were collected: maximum heart rate, minimum heart rate, mean heart rate, and the number of episodes of sustained inappropriate sinus tachycardia (sIST) lasting more than 30 s.

All patients completed the 9-item ASTA symptom scale and the 13-item ASTA HRQoL questionnaire to quantify symptoms and quality of life related to inappropriate sinus tachycardia [13,14], as well as other types of arrhythmias. Patients received SPINOFF^®^ (1 tablet/day) for at least 6 months. After this treatment period, follow-up data were collected (rest 12-lead ECG and 24 h Holter monitoring) and the ASTA questionnaires were re-administered.

The study population was compared with a historical control group of 25 patients receiving ivabradine for the same condition. The ivabradine dosage was titrated from 10 mg/day to 15 mg/day, up to the maximum tolerated dose.

Statistical analysis was performed as follows: quantitative variables were expressed as mean values ± standard deviation. Categorical variables were expressed as percentages (%). The paired t-test (T-score) was used to compare quantitative variables at enrollment (T0) and follow-up (T1), with a *p*-value < 0.05 considered statistically significant.

## 3. Results

This pilot study enrolled 50 recreational athletes (62.5% female, mean age 42 ± 14 years) with frequent recurrences of inappropriate sinus tachycardia. Left ventricular ejection fraction was preserved in all patients (EF 61 ± 4%). Smokers represented 22% of the sample (11/50); 12% (6/50) had hypertension and 34% (17/50) had dyslipidemia.

The historical control group included 25 recreational athletes receiving ivabradine for the same condition (64% female, mean age 45 ± 12 years). Left ventricular ejection fraction was preserved in all control group patients (EF 60 ± 3%). Risk factors for cardiovascular disease were present as follows: smoking (20%—5/25), blood arterial hypertension (16%—4/25), and dyslipidemia (32%—8/25).

**Figure 1:**
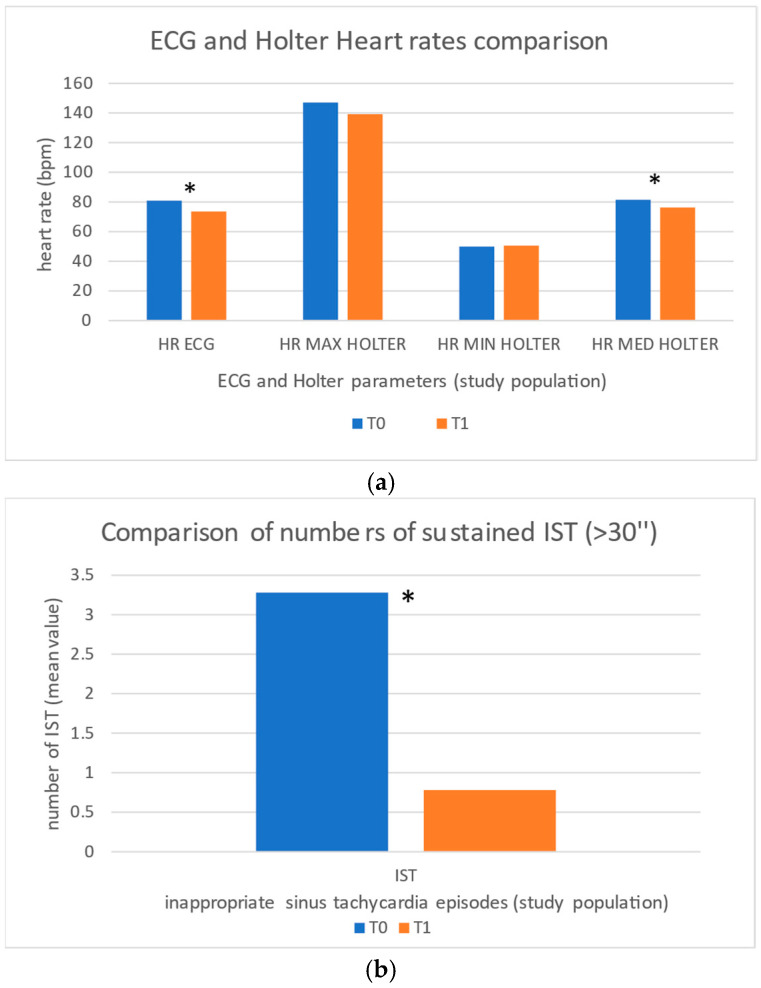
(**a**) Figure shows a comparison of resting ECG heart rate (HR ECG) and Holter ECG findings at enrolment (T0—blue columns) and after treatment (T1—orange columns) among study population. Significant reductions in HR ECG and medium heart rate Holter ECG (HR MED HOLTER) were observed. * represents a statistically significant variation. (**b**) Figure shows a comparison of mean number of sustained (>30”) inappropriate sinus tachycardia (IST) episodes recorded by Holter ECG monitoring, among study population at enrollment (T0—blue column) and after treatment (T1—orange column). * represents a statistically significant variation.

A minimum follow-up period of 6 months was obtained for each patient. The following electrocardiographic findings were observed at T0 and T1 (Figure 1a): resting ECG heart rate was 88.7 ± 12.4 bpm (T0) and 73.6 ± 6.6 bpm (T1) (*p* < 0.00001); Holter average heart rate was 88.4 ± 3.3 bpm and 74.9 ± 4.8 bpm (*p* < 0.0001). Holter ECG maximum heart rate was 147.1 ± 16.7 bpm and 139.2 ± 16.8 bpm, with a non-significant reduction trend (*p* = 0.06). Holter minimum heart rate was 49.9 ± 6.5 bpm and 50.5 ± 6.9 bpm (*p* = 0.33). The number of sustained IST episodes (Figure 1b) significantly decreased from 3.3 ± 1.7 to 0.8 ± 0.8 (*p* < 0.00001).

**Figure 2:**
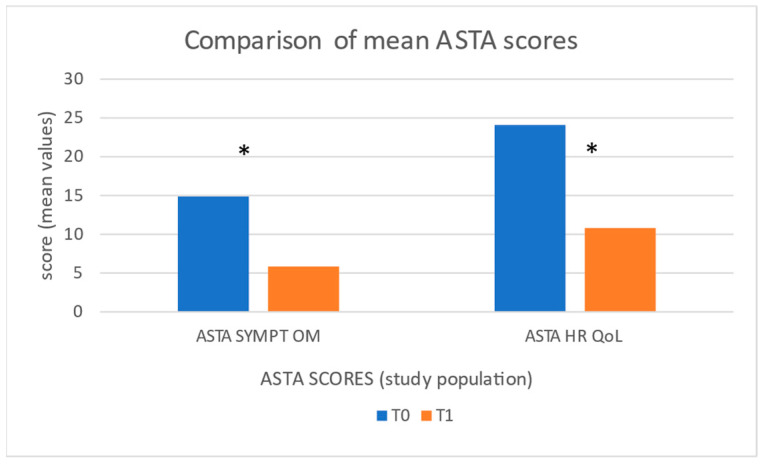
Figure shows a comparison of ASTA score mean values among study population at enrolment (T0—blue columns) and after treatment (T1—orange columns). * represents a statistically significant variation.

The following changes in ASTA scores were observed (Figure 2): the ASTA symptom scale (Table 1a: range: 0–27) decreased from 14.9 ± 2.1 to 5.8 ± 1.4 (*p* < 0.00001), while the ASTA HR QoL (Table 1b: range: 0–39) decreased from 24.1 ± 2.1 to 10.8 ± 2.3 (*p* < 0.00001).

No side effects were recorded during the observation period in the study population; no patient had to discontinue the food supplement due to side effects.

**Figure 3:**
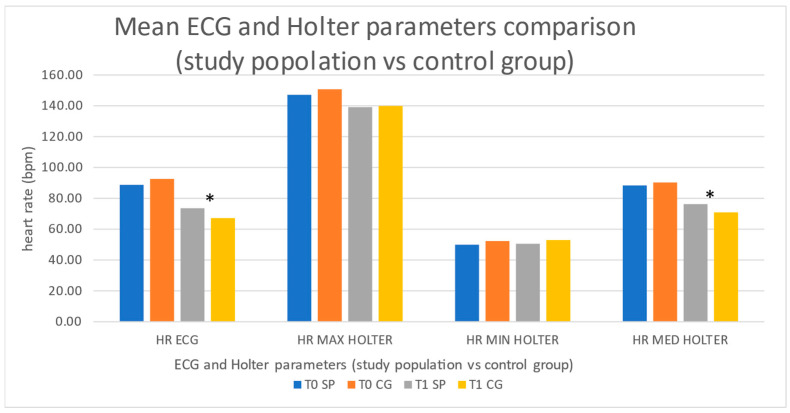
Figure shows mean values of resting ECG heart rate (HR ECG) and Holter ECG findings (max–min—med HR) at enrolment (T0) and after treatment (T1) from study population (SP) compared to historical control group (CG) receiving ivabradine. Blue columns and orange columns represent parameters, respectively, from SP and CG at T0. Gray columns and yellow columns represent parameters, respectively, from SP and CG at T1. When compared to ivabradine, the food supplement treatment showed a lower reduction in resting ECG heart rate and average Holter ECG heart rate. * represents a statistically significant variation (see text).

When compared to the historical control group receiving ivabradine (Figure 3), no statistically significant differences were observed in T0 parameters. After treatment (T1), the ivabradine group showed a statistically more significant reduction in resting ECG heart rate (67.2 ± 5.5 bpm vs. 73.6 ± 6.6 bpm; *p* = 0.02) and Holter ECG average rate (70.9 ± 4.8 bpm vs. 74.9 ± 4.8 bpm; *p* = 0.01) when compared to the study population group; no significant differences in the remaining Holter ECG parameters where observed.

However, 8% of patients in the ivabradine group discontinued treatment due to phosphenes.

## 4. Discussion

Main findings: The present study investigated, for the first time, the use of a novel food supplement (SPINOFF^®^), containing linden, hawthorn, vitamin B1, and melatonin, for the treatment of athletes with inappropriate sinus tachycardia. Our pilot study suggests that this food supplement may play a beneficial role in managing symptoms and improving quality of life in such patients. These clinical effects correlate with significant improvements in resting ECG parameters and Holter ECG findings.

IST is a chronic condition that can significantly impact quality of life. The treatment remains challenging, mainly due to the complex nature of the syndrome and the fact that controlling heart rate does not always alleviate symptoms. In athletes, the treatment can be particularly difficult due to low blood pressure, which contraindicates beta-blockers or other antiarrhythmic medications, and because athletes may be unwilling to take such drugs.

Managing IST includes lifestyle modifications, as well as non-pharmacological and pharmacological interventions. A small study by Fu et al. on 19 patients with IST and postural orthostatic tachycardia syndrome supported the use of exercise training to improve quality of life and maintain upright cardiac output compared to propranolol [15]. The use of beta-blockers in IST is usually ineffective and associated with many adverse effects [16,17]. In a study of 20 patients, metoprolol was less effective than ivabradine in reducing heart rate and improving symptoms [17]. Ivabradine is a promising drug that inhibits the If current, reducing heart rate and improving symptoms. Multiple small studies have reported moderate efficacy [7,8,9,10,11,12,13,14,15,16,17,18]. A multicenter study by Cappato et al. [19] found that ivabradine significantly improved symptoms in 21 patients with IST, eliminating them in approximately half of the patients. However, ivabradine’s long-term efficacy is still not well established.

Finally, radiofrequency ablation of the sinus node has been studied in small populations [20,21]. Despite excellent procedural success rates, tachycardia often recurs from other areas of the sinus node or atrioventricular junction, limiting long-term efficacy. Due to the potential complications (e.g., permanent pacing, phrenic nerve damage, or superior vena cava stenosis), radiofrequency ablation should be reserved for patients who remain symptomatic despite medical treatment and lifestyle modifications [16].

In our preliminary experience, after 6 months of treatment with the food supplement, we observed a significant reduction or, in some cases, complete suppression of symptoms, as well as a notable improvement in quality of life. These clinical benefits correlated with reductions in resting ECG heart rate, maximum and average Holter heart rates, and, most notably, the number of episodes of sustained IST. The exact mechanisms behind these findings remain unclear, but may involve the relaxing (sedative and anxiolytic) effects of linden [11], combined with the antianxiety and cardiovascular effects of hawthorn [12,22,23,24], which could reduce adrenergic stimulation of the sinus node. Furthermore, improved sleep quality, facilitated by melatonin [10], may contribute to reducing stress-induced sinus tachycardia.

While the food supplement showed lower efficacy compared to standard medical treatment (ivabradine), it had the advantage of being better tolerated, with no discontinuations due to side effects, whereas 8% of patients in the ivabradine group stopped treatment due to phosphenes. The lack of known side effects may enhance patient compliance with food supplement therapy compared to antiarrhythmic drugs or beta-blockers.

A limitation of this study is the small sample size and relatively short follow-up period. Therefore, larger-scale randomized studies are needed to confirm these preliminary findings.

## 5. Conclusions

Inappropriate sinus tachycardia (IST) is a chronic condition associated with a significant loss of quality of life. Despite the challenges in medical treatment, our preliminary study suggests that a novel food supplement containing linden, hawthorn, vitamin B1, and melatonin could play a beneficial role in managing symptoms and improving quality of life in patients who refuse or cannot tolerate standard medical therapies. Larger, randomized clinical trials are needed to fully evaluate these findings.

Study Limitations: This study enrolled a small number of patients. Medium- and long-term efficacy data are lacking due to the short follow-up period.

## Figures and Tables

**Table 1:** (**a**) and (**b**) show, respectively, ASTA symptoms and ASTA HR-QoL scores. The first one is composed of 9 items, with possible values ranging from 0 to 27. The second one is composed of 13 items, with possible values ranging from 0 to 39.

**a. ASTA Symptom Scale**					
**Item**	**Item Score Distribution**				
	**No**	**Yes, to a Certain Extent (1)**	**Yes, Quite a Lot (2)**	**Yes, a Lot (3)**	**Missing**
Breathlesness during activity					
Breathlesness even at rest					
Dizziness					
Cold sweats					
Weakness/fatigue					
Tiredness					
Chest pain					
Pressure/discomfort in chest					
Worry/anxiety					
**b. ASTA Health-Related Quality of Life Scale (HR QoL)**					
**Item**	**Item Score Distribution**				
	**No**	**Yes, to a Certain Extent (1)**	**Yes, Quite a Lot (2)**	**Yes, a Lot (3)**	**Missing**
Feel unable to work, study, or carry out daily activities					
Spend less time with family/relatives and friends					
Spend less time with aquaintances					
Avoid planning things you would like to do					
Impaired fisical ability					
Impaired ability to concentrate					
Feel dejected or sad					
Feel irritated or angry					
Experience sleep problems					
Negatively affected sexual life					
Afraid of dying					
Deteriorated life situations					
Feel worried that symptoms will re-occur during arrhythmia-free periods					

## Data Availability

The data presented in this study are available on request from the corresponding author due to privacy reasons.
